# Bilateral risk-reducing mastectomy and reconstruction–A 12-year review of methodological trends and outcomes at a tertiary referral centre

**DOI:** 10.1371/journal.pone.0281601

**Published:** 2023-04-12

**Authors:** Aiman Aslam, Zaki Arshad, Amir Ahmed, Chien Lin Soh, Fawz Kazzazi, John R. Benson, Parto Forouhi, Amit Agrawal, Sarah L. Benyon, Michael Irwin, Charles M. Malata

**Affiliations:** 1 School of Clinical Medicine, University of Cambridge, Cambridge, United Kingdom; 2 Imperial Healthcare NHS Trust, London, United Kingdom; 3 Mason Institute for Medicine, Life Sciences and Law, University of Edinburgh, Edinburgh, United Kingdom; 4 Department of Surgery, Cambridge Breast Unit, Addenbrooke’s Hospital, Cambridge University Hospitals NHS Foundation Trust, Cambridge, United Kingdom; 5 School of Medicine, Anglia Ruskin University, Cambridge and Chelmsford, Cambridge, United Kingdom; 6 Department of Plastic & Reconstructive Surgery, Addenbrooke’s Hospital, Cambridge University Hospitals NHS Foundation Trust, Cambridge, United Kingdom; Erasmus MC Cancer Institute, NETHERLANDS

## Abstract

**Introduction:**

Bilateral risk-reducing mastectomy (BRRM) involves removal of healthy breast tissue to substantially decrease the risk of developing breast cancer in individuals with greater susceptibility due to a strong family history or genetic mutation. This retrospective study evaluates cases of BRRM and associated reconstruction performed at a tertiary centre, with emphasis on mastectomy and reconstructive trends.

**Methods:**

A retrospective review of all BRRM cases performed between January 2010 and May 2022 was conducted, with two separate cohorts corresponding to the earlier (group 1) and later (group 2) portion of the time-period. Data collected included demographics, genetic test results, family history of breast/ovarian cancer, co-morbidities, mastectomy type, reconstruction type, surgical histopathology findings and post-operative complications.

**Results:**

A total of 82 patients (group 1 = 41, group 2 = 41) underwent BRRM. The proportion of nipple-sparing mastectomy increased from 14.6% to 56.1% between the two time periods with a reduction in skin-sparing mastectomies from 75.6% to 20.3% (p<0.001). Of the 80 patients who opted to undergo reconstruction, there was a significant decrease in combined flap-implant reconstructions (19.51% to 0%, p<0.01). Importantly, for implant-only reconstruction, there were significant increases in prepectoral approaches (p = 0.0267) and use of acellular dermal matrix (ADM) (48.15% to 90.63%, p<0.001).

**Conclusion:**

This study documents recent increases in nipple-sparing techniques for BRRM compared to more traditional skin-sparing methods. Concurrently, reconstruction following RRM has become predominantly implant-based without a flap, coinciding with more widespread usage of ADM. This is consistent with national trends towards fewer complex autologous procedures.

## Introduction

Breast cancer is the most common female cancer worldwide, with a reported global incidence of 46.3 per 100,000 women that is continuing to rise [1]. This trend is mirrored in the United Kingdom (UK) where the disease accounts for 30% of female cancer cases [2]. The national incidence of breast cancer increased by 23% between 1993 and 2017 with a current estimate for average lifetime risk for developing breast cancer in the UK of 14.3% [2].

For women at a “high risk” of developing breast cancer (defined as a lifetime risk of 30% or greater when compared to average lifetime risk [2,3]), several primary preventative options are available including surveillance, chemoprevention or bilateral risk-reducing mastectomy (BRRM). BRRM has been shown to reduce the lifetime risk of breast cancer by more than 90% in high-risk populations, as well as decreased mortality when compared to surveillance-only strategies in select patients with genetic mutations [4,5]. Indications for RRM include: strong family history of breast and/or ovarian cancer; presence of mutations in high penetrance genes predisposing to breast cancer (such as BRCA1, BRCA2, TP53, PTEN and PALB2), and atypical proliferative abnormalities on breast histology [6]. Indeed, the presence of a gene mutation is predicted to increase a woman’s lifetime risk of breast cancer from 12% to 72% [7]. Development of formal guidelines for BRCA testing in cases of suspected hereditary cancer as well as increased public awareness of risk-reducing mastectomy have led to an increase in women, seeking genetic testing and subsequently BRRM (especially under 40 years of age) [8,9]. It is increasingly important for clinicians to be familiar with managing risk appropriately in this specific population subset.

Individuals contemplating RRM should consider several factors including post-operative complications, personal perception of body image and sexuality, and negative effects on cosmetic and social outcomes; a process that can be hampered by lack of readily accessible information [10,11]. The optimal mastectomy technique adds to the complexity of decision-making, with both skin-sparing and nipple-sparing techniques offering safety and oncological efficacy in appropriately selected patients [12].

Reconstruction can be offered to mastectomy patients and involves consideration of both timing (immediate versus delayed) and type of reconstruction (implant-based, autologous tissue-based or a combination) [10,13,14]. Furthermore, advances in materials technology and surgical techniques have permitted a greater range of reconstructive options with a notable increased use of acellular dermal matrix (ADM) and prepectoral implant-based breast reconstruction (IBBR) [15]. However, complications following RRM can be influenced by type of reconstruction and it is therefore important to consider both risk-reducing mastectomy and reconstruction in tandem in order to fully appreciate the issues involved.

This study aimed to review cases of BRRM (with or without reconstruction) undertaken in a tertiary referral centre between 2010 and 2022, with recognition of two distinct time periods. The primary objective was to undertake a comparison of results between earlier and later time periods, with a secondary objective characterising early surgical complications. Key trends in terms of techniques for risk-reducing mastectomy and associated reconstruction will be identified, and provide clinicians with a broad overview of evolving practice within breast surgery, thereby aiding the decision-making process.

## Methods

### Patient sample

A retrospective review of patients undergoing bilateral risk-reducing mastectomy between January 2010 and May 2022 from a single tertiary referral centre was performed. Specific approval from the IRB/Ethics Committee was not required for this retrospective project as all data had been collected already as part of routine clinical patient care, and therefore the work falls under the remit of Audit and Clinical Governance Service Improvement Projects. IRB approval is not required for such projects which do not alter the care of the patients in the study and registration numbers are not issued by the ethics committee for such projects that do not fall under their remit. Approval for undertaking this retrospective project was granted by the hospital’s audit and clinical governance department, and all ethical principles were followed in the creation of this manuscript. The hospital project ID is 3160 and hospital project registration number is PRN9160. Patients were identified from the prospectively maintained departmental breast reconstruction register together with operating theatre records. Data were then extracted from an archival electronic hospital records system for cases diagnosed prior to October 2014 and EPIC Systems Corporation for cases thereafter. All patients undergoing risk-reducing mastectomy were included, irrespective of any breast reconstruction. As this data were collected as part of routine clinical care provided to patients, consent did not need to be sought specifically for this retrospective paper. There were no patient identifying data. All patients were counselled and informed on the benefits versus risks of treatment options as part of ongoing routine care.

### Data collection

Data collected included demographics, body mass index (BMI), pre-operative genetic test results, family history of breast or ovarian cancer, comorbidities, performance status (classified by the American Society of Anaesthesiologists [ASA] score), and smoking status at time of surgery. The following factors were also recorded: 1) type of mastectomy (simple, skin-sparing, nipple-sparing, or skin-reducing) 2) reconstruction technique (implant-based, solely autologous, or implant-assisted flaps) 3) laterality of RRM (unilateral or bilateral) 4) postoperative histopathology 5) 3-month post-operative complications (rates of flap/ implant loss, necrosis, infection and reoperation/ readmission) 6) any de novo cancer occurrence 7) whether risk-reducing salpingo-oophrectomy had been performed and 8) the timing of this in relation to the BRRM. Readmission was defined as any inpatient stay related to the surgical procedure after primary discharge. Reoperation was defined as any major surgical procedure requiring unplanned return to the operating room. Final follow-up was taken to be the most recent oncology, breast surgery or plastic surgery appointment with a clinician.

### Statistical analyses

Statistical analysis was performed using IBM SPSS statistical software (v28.0; IBM Corp., Armonk, New York, USA). The cohort was divided into two groups with group 1 corresponding to patients undergoing surgery berween January 2010 and December 2014 and group 2 between January 2015 and April 2022. This turning point between the two groups was an arbitrary time point that allowed for coverage of similar periods of time in both groups, and therefore observation of trends over time, as well as coinciding with the adoption of our totally electronic patient record and a lowering of the national threshold for genetic testing from a likelihood of 20% to 10%. Pearson’s chi-squared test was used for comparison of categorical variables with post-hoc analysis performed for sub-group analysis. Fisher’s exact test was used where expected counts were below five. For comparing continuous data, analysis of normality using the Kolmogorov-Smirnov and Shapiro-Wilks tests alongside visual inspection of normal Q-Q plots was conducted, with the independent T-test used for normally distributed data and the Mann-Whitney U test for nonparametric data. A value of p<0.05 was considered to be significant.

## Results

A total of 82 BRRM patients (164 breasts) were identified amongst whom 41 (50%) underwent surgery between January 2010 and December 2014 (group 1; mean age = 38 years) and 41 (50%) patients between January 2015 and April 2022 (group 2; mean age = 39.9 years). Characteristics for each of these two groups are shown in **Table 1**. The mean follow-up period was 39.12 months (2–136 months).

**Table 1:** Summary of patient characteristics.

Variables	Group 1 [n = 41]	Group 2 [n = 41]	p-value
**Mean age, years (range)**	38 (21–57)	39.88 (24–60)	0.53*
**Mean Body Mass Index (range)**	27.73 (21–33)	25.58 (18.67–33.7)	
**Smoking status, n (%)**			0.26†
Smoker	4 (9.76)	5 (12.20)	
Ex-smoker	8 (19.51)	14 (34.15)	
Never smoker	29 (70.73)	22 (53.66)	
**Past medical history, n (%)**			
Non-cancer comorbidities			0.90†
*Nil*	27 (65.85)	27 (65.85)	
*1*	9 (22.0)	7 (17.07)	
*2*	3 (7.32)	4 (9.76)	
*3+*	2 (4.88)	3 (7.32)	
**Family history of breast/ ovarian cancer, n (%)**			0.51†
Nil family history	5 (12.20)	3 (7.32)	
≥ 1 first-degree relative with breast/ovarian cancer	32 (78.05)	31 (75.61)	
No first-degree relatives but ≥ 1 second-degree relative	4 (9.76)	7 (17.07)	
**ASA score, n (%)**			0.66†
1	22 (53.66)	26 (63.41)	
2	18 (43.90)	14 (34.15)	
3	1 (2.44)	1 (2.44)	

* = Independent t-test

† = Chi-square test.

There was no significant difference in age (p = 0.53), frequency of non-malignant comorbidities (p = 0.90), ASA score or smoking status (p = 0.26) between the two groups. For ex-smokers, the mean length of time that had passed between cessation of smoking and BRRM was 2 years (range: 1–3 years) for group 1 and 5.81 years (range: 1–20 years) for group 2. Of note, in Group 1 there was one patient with Hodgkin’s mantle cell lymphoma and in Group 2, there were four patients with a history of malignancy: one ovarian cancer, one Ewing’s sarcoma, one cervical cancer and one serous tubular intraepithelial carcinoma.

**Fig 1:**
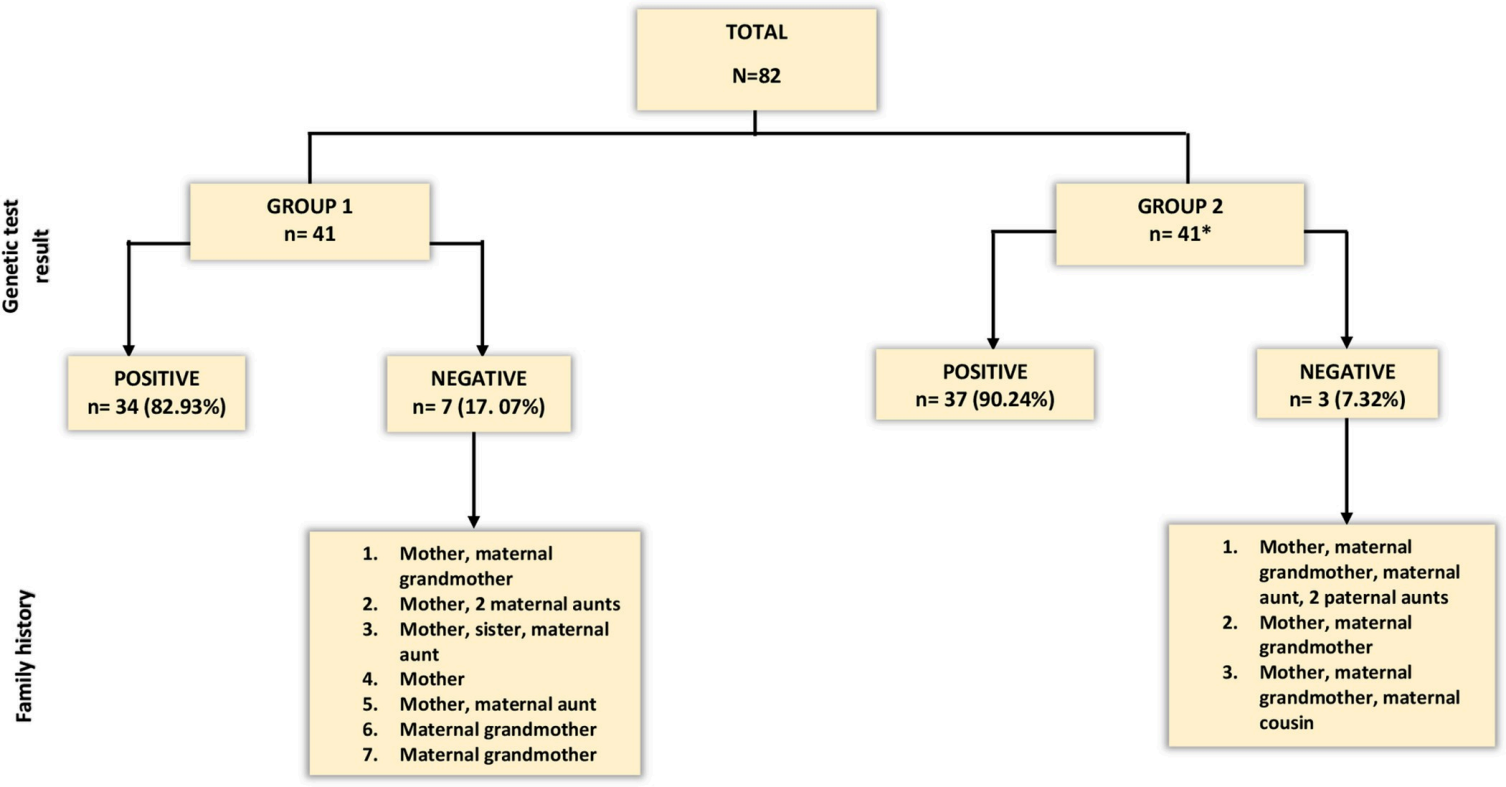
Genetic test outcome for all patients and family history breakdown for patients with negative genetic results. A positive family history was defined as at least one first or second degree relative with breast or ovarian cancer. *In Group 2, one patient was not genetically tested and did not have positive family history. However she was a candidate for prophylactic surgery due to a significant past medical history of Ewing’s sarcoma of the right lung, treated by radiotherapy, surgery and post-operative chemotherapy.

All patients except one underwent genetic testing prior to risk-reducing surgery (**Fig 1**). **Fig 1** displays results of genetic testing; all patients with a negative genetic test result had a positive family history. Of the 34 patients who tested positive for a pathogenic variant in group 1, 21 (61.76%) had a BRCA1 and 13 (38.24%) had a BRCA2 mutation. Amongst the 37 patients with a positive genetic test result in group 2, there were 18 (48.7%) cases of BRCA1 and 19 (51.4%) cases of BRCA2 mutations.

**Table 2** shows the proportion of each type of BRRM performed. Each patient received the same type of mastectomy when performed bilaterally, be this skin-sparing, nipple-sparing, skin-reducing pattern or simple mastectomy. There were notable changes in the type of mastectomy performed over the study period, with subgroup analysis identifying a significant decrease in the proportion of skin-sparing mastectomy (75.6% to 29.3%, p<0.001) and a corresponding increase in nipple-sparing mastectomy (14.6% to 56.1%, p = 0.0016).

**Table 2:** Summary of bilateral risk-reducing mastectomy procedures performed throughout the study period.

Variables, n (%)	Group 1 [n = 41]	Group 2 [n = 41]	p-value
**Type of mastectomy**			*<0*.001*
Skin-sparing	31 (75.61)	12 (29.27)	*<0*.*001†*
Nipple-sparing	6 (14.63)	23 (56.10)	*0*.*0016†*
Skin-reducing	4 (9.76)	4 (9.76)	1.00÷
*Wise pattern*	4 (9.76)	2 (4.88)	
*LeJour pattern*	0	2 (4.88)	
Simple	0 (0)	2 (4.88)	0.58†

* = Fisher’s exact test

† = Post-hoc analysis with Bonferroni correction (α = 0.00625).

All breast reconstructions in this study cohort were perfomed as an immediate procedure with most patients opting for breast reconstruction (80/82, 97.56%) (**Table 3**). The proportion of each type of reconstruction was found to differ significantly between the two groups (p = 0.0104). Sub-group analysis revealed no significant increase in the proportion of implant-based breast reconstruction from the earlier to the later time period (IBBR) (65.9% to 78.1%). However, there was a statistically significant decrease in combined implant-assisted latissimus dorsi flap reconstruction (19.5% to 0%, p<0.01) when comparing the later with the earlier time period.

**Table 3:** Reconstructive surgery data in patients undergoing BRRM over the entire study period.

Variables, n (%)	Group 1 [n = 41]	Group 2 [n = 41]	p-value
**No reconstruction performed**	0 (0)	2 (4.88)	0.4938*
**Reconstruction performed**	41 (100)	39 (95.12)	
**Reconstruction type**			*0*.0104*
**IMPLANT ONLY**	27 (65.85)	32 (78.05)	0.110**÷**
***Type***			*0*.0267*
Subpectoral	27	26	
Prepectoral	0	6	
***ADM used***	13	29	*<0.001†*
**AUTOLOGOUS ONLY**	6 (14.63)	7 (17.1)	0.689**÷**
***Type***			
DIEP flap	5	7	
SIEA flap	1	0	
**COMBINED FLAP-IMPLANT**	8 (19.51) ^**1**^	0 (0)	*0*.*00373***÷**

RRM = Risk-reducing mastectomy; LD = Latissimus dorsi

DIEP = Deep inferior epigastric artery perforator

SIEA = Superficial inferior epigastric artery

^**1**^ = all combination reconstructions consisted of LD flaps and 7/8 with expandable implant, 1/8 with fixed volume implant

* = Fisher’s exact test

† = Chi-square test

÷ = Post-hoc analysis with Bonferroni correction (α = 0.0083).

Implant reconstruction was further stratified into subpectoral or prepectoral implant placement. No cases of prepectoral reconstruction were performed during the earlier time period (group 1), but 6/32 implant-only reconstructions were documented as prepectoral between 2015 and 2019 (p = 0.0267) (group 2). The numbers of exclusively autologous flap reconstruction were similar for groups 1 and 2.

A notable observation was the significant increase in use of ADM between the two time periods, with just under half (13/27, 48.2%) of implant-only reconstructions having ADM in group 1, compared with the majority of implant-only reconstructions in group 2 (29/32, 90.6%, p<0.001).

**Table 4** summarises post-operative outcomes for all patients. A total of 9/41 (21.95%) patients experienced at least one complication in group 1, compared to 10/41 (24.4%) in group 2 (p = 0.59). Of the 23 patients who had nipple-sparing mastectomy in group 2, 4 (17.39%) developed nipple necrosis within the first 3 months after surgery.

**Table 4:** Summary of post-operative characteristics including post-operative complications, Clavien-Dindo score, and post-operative histopathology.

Variables	Group 1 [n = 41]		Group 2 [n = 41]		p-value
**Complication type, n (%)**	**Total eligible patients**	**Patients with complication**	**Total eligible patients**	**Patients with complication**	
Implant loss	35^**1**^	0 (0)	32^**1**^	2 (6.25)	
Readmission	41	6 (14.63)	41	10 (24.39)	
Reoperation	41	6 (14.63)	41	8 (19.51)	
Infection	41	3 (7.32)	41	5 (12.20)	
Flap loss	14^**2**^	0 (0)	7^**2**^	0 (0)	
Nipple necrosis	6	1 (16.67)	23	5 (21.74)	
Overall complication rate	41	9 (21.95)	41	10 (24.39)	0.794*
**Clavien-Dindo score, n (%)**	**Group 1**		**Group 2**		0.693†
0	24 (58.54)		24 (58.54)		
1	11 (26.83)		8 (19.51)		
2	3 (7.32)		3 (7.32)		
3	3 (7.32)		6 (14.63)		
**Post-operative histopathology, n (%)**	**Group 1**		**Group 2**		0.581†
DCIS	3		1		
LCIS	1		1		
Atypia	0		2		
No atypia or in-situ/invasive malignancy	37		37		

^1^ including implant-only and flap-implant combinations

^2^ including autologous-only and flap-implant combinations * = Chi-square test

† = Fisher’s exact test. The overall complication rate for each group is determined by the number of patients who experienced at least one of the listed complications.

Almost half of women had also undergone bilateral risk-reducing salpingo-oophorectomy by the end of the study period (group 1, 19/41 (46.3%); group 2, 18/41 (43.90%)) with oophorectomy preceding BRRM in 10 and 15 patients for groups 1 and 2 respectively. There were no cases of breast or ovarian cancer during the period of follow-up for this patient cohort.

## Discussion

This single-centre study reviews BRRM and reconstruction performed over a 12-year period at a UK tertiary referral centre. Several key trends in both risk-reducing mastectomy and breast reconstruction are highlighted. In addition, it is reassuring that no cases of breast cancer appeared during follow-up.

### Indications for BRRM

BRRM can reduce breast cancer risk by more than 90% in patients with BRCA gene mutations and/or family history and is the most effective prophylactic therapy against breast cancer development [16,17]. Inherited mutations in high penetrance breast cancer susceptibility genes such as BRCA1 and BRCA2 are associated with relatively high lifetime breast cancer risk [6]. In the present study, all but one patient underwent genetic testing, with more than three-quarters (86.59%) testing positive for a mutation. However, it should be noted that whilst genetic testing can exclude carriage of a known familial gene mutation, limitations of current technologies and intrinsic genetic test sensitivity (approximately 85%) prevent some mutations from being detected and any prophylactic intervention can only be based on family history of breast cancer [18,19]. The small number of patients in the present study without an identified mutation had a strong family history of breast cancer, and were therefore appropriate candidates for BRRM (**Fig 1**) [6].

### Increased use of nipple-sparing mastectomy (NSM)

A nipple-sparing approach to mastectomy has historically been associated with concerns about risk of nipple-areolar-complex (NAC) necrosis and de novo cancer in residual tissue. However, growing evidence for its oncological safety combined with cosmetic appeal and positive psychological adjustment has led to increasing popularity of NSM as a prophylactic procedure [20,21]. The current study highlights a significant increase in rates of NSM from approximately one in seven (14.6%) for the early period to more than half (56.1%) of all BRRMs for the second time period. This is consistent with an upwards trend internationally [20]. Furthermore, there is no evidence from the current study for any increase in de novo breast cancer which therefore supports current literature on the safety of NSM for BRRM [22]. Although candidacy for NSM is usually predicated on a patient having relatively small breasts without ptosis, eligibility criteria in terms of breast size and degree of ptosis have expanded in recent years, with a variety of incisions developed to manage ptosis and enable skin reduction if necessary [23]. Of the 29 patients who underwent NSM across the entire study period, six developed necrosis involving one or both nipples that required surgical debridement (20.7%)–this is a similar figure to other published reports although lack of standard methods for assessment of necrosis has resulted in a wide range of reported rates [24–26]. Smoking is an established risk-factor for NAC necrosis due to adverse effects on macro and microvasculature [26]. Two-thirds (4/6) of patients with NAC necrosis in this study were ex-smokers; clinician-led encouragement and public health initiatives, may reduce the chance of necrotic complications if smoking is avoided or curtailed prior to surgery. Furthermore, the type and length of incision for NSM influence risk of necrosis and therefore both patient and surgical factors need to be carefully assessed when determining candidacy for NSM [26].

### Reconstruction and ADM

This study reveals a significant decrease in flap-implant combined reconstruction and is consistent with nationwide trends documenting a sharp decline in combined procedures, particularly implant-assisted LD flaps [27,28]. This may partly be attributed to the advent of ADM and mesh devices. These act as biological scaffolds that become incorporated into host tissue and provide additional tissue coverage for implant-only reconstruction with more effective healing and reduction in rates of peri-implant inflammation and capsular contracture [29]. Indeed, this preference for implant reconstruction without autologous tissue has also has been encouraged by availability of a more comprehensive selection of implants that permit a more tailored surgical approach and “individualisation” of outcomes [30].

### Increased use of the prepectoral approach in implant-based reconstruction

Although a subpectoral approach for implant placement has been conventional for breast reconstruction, ADMs have permitted the alternative option of prepectoral placement of implants that are encased by “mesh”. This approach reduces animation deformity and post-operative pain scores by leaving the pectoralis major muscle undisturbed, while simultaneously addressing problems of inadequate soft tissue support for implants placed subcutaneously. Current cosmetic outcomes and complication rates appear comparable to subpectoral placement although there is a lack of long-term efficacy data on the advantages of prepectoral reconstruction [31,32].

The current study found a significant increase in prepectoral breast reconstruction over time, although numbers remain low (only 6/26 for group 2). This is partly explained by stringent patient selection criteria with clear contraindications to pre-pectoral IBBR that include: poor skin quality/vascular compromise of skin flaps, poorly controlled diabetes, active tobacco use, previous or planned radiotherapy and presence of invasive tumour [33]. Younger patients are more likely to be physically active and are more often considered for a prepectoral approach [32]. There are a dearth of studies examining use of prepectoral implants for reconstruction after BRRM. Two studies have reported high levels of patient satisfaction and low complication rates [34,35]. This upwards trend in use of prepectoral reconstruction is likely to continue for two important reasons. Firstly, an increasing number of cases of risk-reducing mastectomy are being performed in younger patients, with greater absolute benefit for life-expectancy when RRM is performed at an early age [32]. Secondly, *Marks et al* conducted a survey revealing that almost 50% of American plastic surgeons used prepectoral techniques in “all” or “most” cases of breast reconstruction. Moreover, prepectoral IBBR was more likely to be adopted by surgeons with less than 15 years’ experience [36], suggesting that contemporaneously trained surgeons are more receptive to and proficient with this technique.

### Limitations and COVID-19 considerations

There are some limitations to this study. Firstly, the follow-up range is wide, with three patients undergoing surgery in the final year of the study period. Sample size is relatively small and longer follow-up as well as larger sample sizes are required to more accurately determine rates of de novo breast cancer development and to characterise long-term complications such as development of capsular contracture more accurately.

Furthermore, several different breast surgeons have performed risk-reducing breast surgery over the decade in question and this may be reflected in mastectomy trends (especially for NSM). However, trends in reconstruction are less likely to be attributed to staff changes in this study as the same three plastic surgeons performed the majority of reconstructions up until 2015 after which one oncoplastic breast surgeon undertook both the extirpative and reconstructive components of surgery.

The COVID-19 pandemic limited the practice of BRRM and reconstruction due to restricted operative capacity, prioritisation of urgent cancer cases and cancellation of elective cases. As such, only five patients had surgery between January 2020 and the end of the study period (May 2022), with two cases of conventional mastectomy without reconstruction during this later time period. It would be useful to characterise the future trends in reconstructive practices post-pandemic with recent events having accelerated a decline in autologous tissue reconstruction [37].

Improvements in surgical techniques and materials technology should focus on maximising risk reduction while optimising functional, aesthetic and quality of life outcomes.

### Conclusion

The current study highlights the changing landscape of bilateral risk-reducing mastectomy and reconstruction, reflecting a national shift towards more nipple-sparing approaches and fewer complex autologous procedures. When considering the range of techniques within both mastectomy and associated reconstruction, several permutations of surgical options can be offered to patients. As breast surgery continues to evolve, it is important to evaluate specific trends such as more conservative forms of mastectomy as well as novel techniques and devices for breast reconstruction to ensure optimal patient care and levels of satisfaction.

## Supporting information

S1 File Data set for study. (XLSX)
